# The successful transfer of a modern CHO fed-batch process to different single-use bioreactors

**DOI:** 10.1186/1753-6561-7-S6-P32

**Published:** 2013-12-04

**Authors:** Sebastian Ruhl, Ute Husemann, Elke Jurkiewicz, Thomas Dreher, Gerhard Greller

**Affiliations:** 1 Sartorius Stedim Biotech GmbH, D-37079 Göttingen, Germany

## Introduction

Nowadays, single-use bioreactors are widely accepted in pharmaceutical industry. This is based on shorter batch to batch times, reduced cleaning effort and a significantly lower risk of cross contaminations [1,2]. One large field of the application of single-use bioreactors is the seed train cultivation of mammalian cells [1]. The focus is further extended to perform state of the art fed-batch production processes in such bioreactors. In this study an industrial proven CHO fed-batch process is established in different single-use and reusable bioreactors.

## Materials and methods

### Cell line, medium and process strategy

For the fed-batch process the cell line CHO DG44 (Cellca, Germany) secreting human IgG1 was used. SMD5 medium (Cellca, Germany) was prepared for the seed train and PM5 medium (Cellca, Germany) as a basal medium for the fed-batch culture. The feeding procedure comprised the addition of three different feeds (feed medium A, feed medium B and concentrated glucose solution). After a 3 day batch phase, the 14 day fed-batch phase started. The automated discontinuous bolus feed of feed media A and B was supplemented by the glucose feed solution to keep the glucose concentration above 3 g/L.

### Bioreactors

The process was initially developed in a 5 L stirred glass bioreactor therefore the BIOSTAT^® ^B with a UniVessel^® ^5 L was considered as a reference. Single-use bioreactors involved in this study were the stirred tank reactor BIOSTAT^® ^STR 50 L with a CultiBag STR 50 L and the rocking motion bioreactor BIOSTAT^® ^RM 50 optical with CultiBag RM 50 L.

### Process transfer

The used bioreactors were characterized in terms of process engineering [3]. Due to different agitation and gassing principles present in the BIOSTAT^® ^STR and RM the k_L_a and mixing times were chosen as a scale-up criteria. The process conditions were specified to meet a k_L_a-value of > 7 h^-1 ^[4] and a mixing time of < 60 s [5].

### Sampling procedure

A daily sampling procedure was performed before the bolus feed. Metabolites like glucose and lactate were analyzed by the Radiometer ABL800 basic (Radiometer, Germany). Viable cell density (VCD) and viability were determined by the Cedex HiRes (Roche Diagnostics, Germany).

## Results

The process transfer is considered successful, if comparable cellular proliferation activities and product titers are obtained.

**Figure 1:**
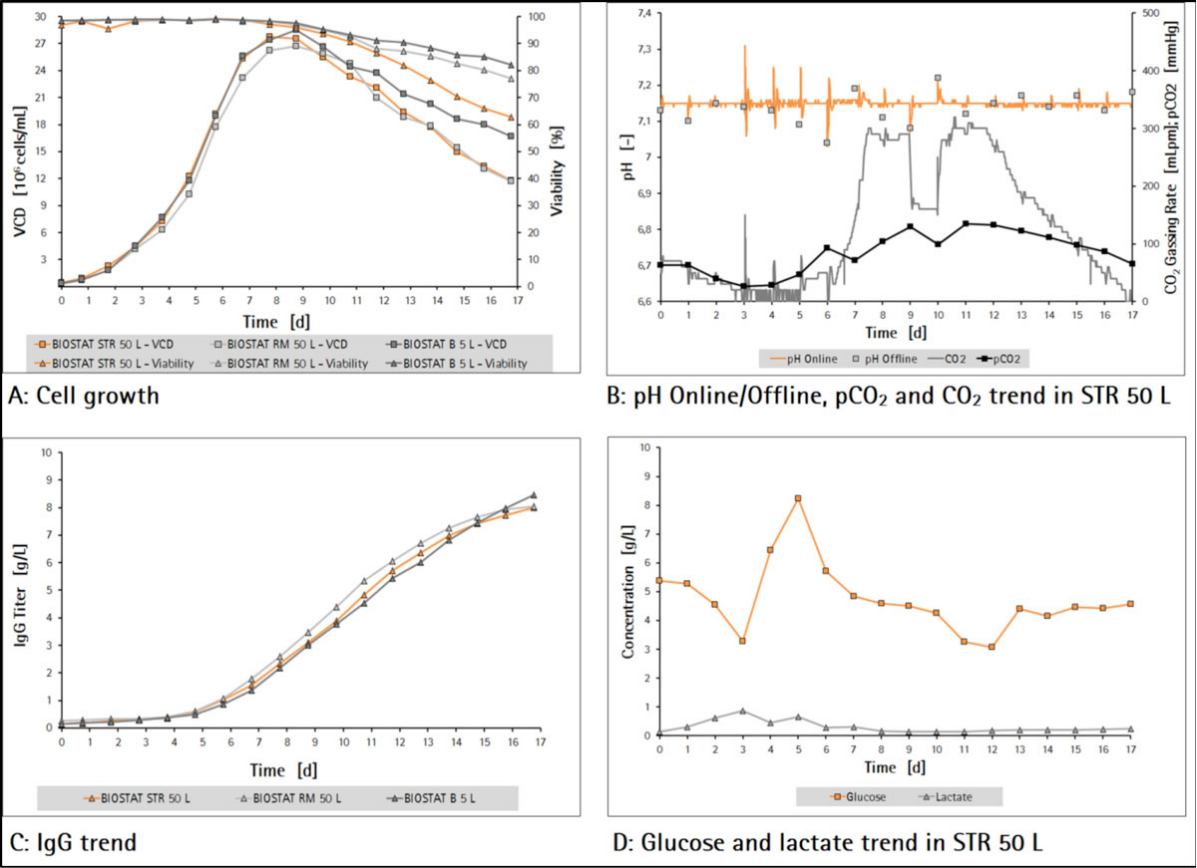
**Process trends**.

The initial viable cell density in all systems was 0.3 - 0.4 × 10^6 ^cells/mL. At the start of the fed-batch phase a viable cell density of 4 - 5 × 10^6 ^cells/mL could be achieved. As seen in Figure 1A the viable cell density peak of 27 - 28 × 10^6 ^cells/mL was reached in all systems after 8 - 9 days. At the point of harvest after 17 days viable cell densities between 12 - 17 × 10^6 ^cells/mL and viabilities of 57 - 82% were reached. The cell broth was harvested for further downstream operations.

A well-controlled pH value is essential for a reproducible cell proliferation. As seen in Figure 1B exemplarily shown for the BIOSTAT^® ^STR 50 L small peaks occurred due to the daily addition of feed medium B (pH 11). The offline measured pCO_2 _trend shows a constant decrease during the batch phase followed by an increase during the fed-batch phase with a maximum value of 135 mmHg.

Shown in Figure 1D glucose concentration could be kept above 3 g/L in the fed-batch phase. Lactate had a peak accumulation of 0.9 g/L at the end of the batch phase and remained at low value afterwards.

The product yield in all cultivations was comparable to the reference systems and exceeded 8 g/L IgG (Figure 1C).

## Conclusion

The high cell density CHO fed-batch process with industry relevant titers was successfully transfer from a reference bioreactor to a variety of single-use bioreactor systems.

The k_L _a and mixing time were suitable as a scale-up criteria for systems with different agitation principles.

**Table 1:** Bioreactor Setup and Process Parameters

BIOSTAT^®^	RM 50 L	STR 50 L	B 5 L
Gassing principle	Overlay	Ring Sparger	

Sensors	Single-use optical patches		Reusable probes

Working volume [L]	25	50	5

Initial volume [L]	13	26	2.6

pH set point	7.15		

pH control	CO_2 _gassing		

pO_2 _set point	60% sat.		

pO_2 _control	Multi stage cascade comprising N_2_, Air, O_2 _- gassing		

Agitation [rpm]	30 @ 10° rocking angle	150	400
